# Effect of yoga on self-rated visual discomfort in computer users

**DOI:** 10.1186/1746-160X-2-46

**Published:** 2006-12-03

**Authors:** Shirley Telles, KV Naveen, Manoj Dash, Rajendra Deginal, NK Manjunath

**Affiliations:** 1 Swami Vivekananda Yoga Research Foundation, No. 19, Eknath Bhavan, K.G. Nagar, Bangalore 560 019, India

## Abstract

**Background:**

'Dry eye' appears to be the main contributor to the symptoms of computer vision syndrome. Regular breaks and the use of artificial tears or certain eye drops are some of the options to reduce visual discomfort. A combination of yoga practices have been shown to reduce visual strain in persons with progressive myopia. The present randomized controlled trial was planned to evaluate the effect of a combination of yoga practices on self-rated symptoms of visual discomfort in professional computer users in Bangalore.

**Methods:**

Two hundred and ninety one professional computer users were randomly assigned to two groups, yoga (YG, n = 146) and wait list control (WL, n = 145). Both groups were assessed at baseline and after sixty days for self-rated visual discomfort using a standard questionnaire. During these 60 days the YG group practiced an hour of yoga daily for five days in a week and the WL group did their usual recreational activities also for an hour daily for the same duration. At 60 days there were 62 in the YG group and 55 in the WL group.

**Results:**

While the scores for visual discomfort of both groups were comparable at baseline, after 60 days there was a significantly decreased score in the YG group, whereas the WL group showed significantly increased scores.

**Conclusion:**

The results suggest that the yoga practice appeared to reduce visual discomfort, while the group who had no yoga intervention (WL) showed an increase in discomfort at the end of sixty days.

## Background

Nowadays most people have some contact with computers either at work or at home. This change has been associated with an increase in complaints of a number of health problems associated with working at visual display terminals (VDTs) [1]. Eye problems are the single most common complaints [2]. The main visual symptoms which VDT users report are eyestrain, irritation, tired eyes, a burning sensation, redness, blurred vision, and double vision [2-5]. The symptoms collectively constitute computer vision syndrome [6]. The main contributor to the symptoms of computer vision syndrome appears to be 'dry eye'.

These symptoms are widely recognized as temporary, however the individual does experience considerable discomfort [7]. Reducing visual discomfort appears to improve productivity at work [8]. This was indirectly inferred, as adding regular breaks to the work schedule improved the efficiency between breaks and compensated for the extra time spent in breaks. Apart from breaks other options which have been tried to reduce discomfort are modifying the computer location, the lighting and reflection, increasing humidity, the use of artificial tears [9] or certain eye drops [10].

Yoga is an ancient Indian science which includes the practice of specific postures, cleansing practices, regulated breathing and meditation [11]. A combination of yoga practices reduced symptoms of visual strain in persons with progressive myopia [12]. Among software development organizations worldwide, several are in Bangalore city [13]. Hence the present randomized controlled trial was planned to evaluate the effect of a combination of yoga practices on self-rated symptoms of visual discomfort in professional computer users in Bangalore.

## Methods

### Participants

The participants were 291 persons working in a software company in Bangalore, India. There was no attempt to calculate the sample size when planning the study. However based on the effect size obtained in the present study [0.66 with 0.9 power to detect a significant difference at alpha level 0.05], 50 subjects were required for each group while in fact at baseline there were 146 subjects in the yoga group and 145 in the control group.

All of them used a computer for at least 6 hours each day, for 5 days in a week. Persons of both sexes participated in the trial and their ages ranged between 21 and 49 years. The participants were screened to exclude those who: (i) had consulted a specialist for their visual symptoms, (ii) had uncorrected errors of refraction, (iii) had clinical conditions such as Sjögren's syndrome or kerato-conjunctivitis sicca and (iv) used medication associated with drying of the eyes (e.g., anti-histaminics). None of the participants had to be excluded based on these criteria. The details of the study were described to the participants and their consent to participate was obtained. The project was approved by the ethics committee of the yoga research foundation and had the approval of the human resource department of the software company.

### Design of the study

291 participants were randomized prior to assessment as two groups using a standard random number table by the researchers responsible. The two groups were then designated as (i) intervention (i.e., yoga, n = 146) and (ii) wait list control (n = 145) by an office assistant from the software company who had no other role in the study. The yoga (YG) and wait list control (WL) groups were comparable with respect to age (group average (± S.D.) 32.8 (± 8.6) years and 31.9 (± 10.2) years, respectively) and gender-distribution (11 females in YG group and 13 in WL group).

**Figure 1:**
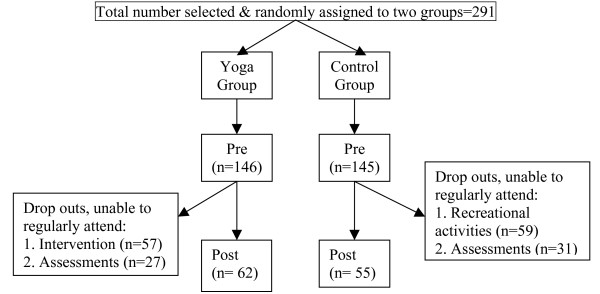
Trial profile of the randomized controlled study.

Both groups were assessed at baseline and after 60 days. During the 60 days the YG group practiced yoga for an hour per day, for five days in a week. While the YG group practiced yoga the WL group spent the time in the recreation center of the software company where 60 percent of them talked to their friends, 12 percent spent time playing indoor games, 12 percent worked out in the gym and 16 percent watched television. The WL group had already been spending this time each day doing the same activities and hence during the 60 day period they were following their usual routine. During the 60 days there were 84 drop outs from the trial in the YG group and 90 from the WL group. The large number of drop outs was mainly due to the fact that the participants had demanding work schedules which interfered with their participating in: (i) the intervention (YG group) or recreational activities (WL group) and/or (ii) the assessments (both groups). To be considered as regular in their participation the YG group had to have a minimum of 38 days of attendance during the 60 day period. The trial profile is given in Figure 1.

### Assessments

Visual discomfort including dryness, irritation, burning, redness, photo-sensitivity and possible remedial measures (e.g., the use of lubricating eye drops) were determined using a questionnaire [14]. It had 12 items, each of which had 4 possible choices. These were: (i) absent, (ii) rarely present (meaning one or two days per week), (iii) often (meaning more than two days per week) and (iv) continuous. The symptoms were considered during the week before assessment and the four alternatives (i-iv) were graded as '0', '1', '2' and '3', respectively. The person who administered the questionnaire and scored the response sheets was not aware to which group the subjects belonged.

### Intervention (yoga)

The 60 minute yoga program included yoga postures (*asanas*, 15 minutes), regulated breathing (*pranayamas*, 10 minutes), exercises for the joints (*sithilikarana vyayama*, 10 minutes), visual cleansing exercises (*trataka*, 10 minutes), and guided relaxation (15 minutes).

The practice of *trataka *involves two sets of eye exercises. (i) Shifting the gaze (by moving the eyes alone) in eight directions. During this exercise, practitioners are asked to use their right thumb (and when gazing to the left, their left thumb) as a cue to direct their gaze. The directions are up, down, up to the left, down to the left, up to the right, down to the right and rotation of the eyes clock-wise and anti clock-wise. (ii) During the second exercise, practitioners gaze at a flame placed at eye level without blinking. While gazing at the flame, practitioners are instructed to focus their gaze on the flame and subsequently defocus while keeping their gaze on the flame. Throughout the practice practitioners should sit upright and should avoid moving their head to shift their gaze.

These techniques were selected either because previous research showed that they reduced physiological arousal [15,16] or based on our unpublished clinical observations.

### Data analysis

The data were analyzed using SPSS Version 10.0. Repeated measures analyses of variance (ANOVA) were carried out with one Between-subjects factor, viz., Groups (with two levels, i.e., YG and WL groups) and one Within-subjects factor, viz., Assessments (with two levels, i.e., baseline and day 60). *Post-hoc *analyses for multiple comparisons between mean values were done with Bonferroni adjustment.

## Results

291 participants attended their respective interventions [yoga and control] in three blocks across 18 months from October 2004 to April 2005.

The repeated measures analyses of variance (ANOVA) showed a significant difference between YG and WL groups (F = 15.369, DF = 1,115, P < .001). There was no significant difference between assessments taken at baseline and on day 60. The interaction between groups and assessments was significant (F = 178.607, DF = 1,115, P < .001), suggesting that the two factors (groups, assessments) were not independent of each other.

*Post-hoc *assessments with multiple comparisons of mean values showed a significant decrease in scores of self-rated visual discomfort for the YG group on day 60 compared to baseline (P < .001). In contrast, there was a significant increase in scores of self rated visual discomfort for the WL group on day 60 compared to baseline (P < .001).

The groups mean values with 95% C.I. are given in Table 1. The details of the ANOVA are given in Table 2.

**Table 1:** Scores of the questionnaire for visual discomfort for yoga and control groups at baseline [BL] and day 60.

**Descriptive values**	**YOGA [n = 62]**		**CONTROL [n = 55]**	
	**BL**	**Day 60**	**BL**	**Day 60**

**Mean (95 % C.I.)**	1.03 (.91–1.15)	0.7*** (.58–.94)	1.05 (.89–1.21)	1.5*** (1.34–1.66)

*** P < .001, post-hoc test for multiple comparisons

**Table 2:** Analysis of variance for scores in the 'Dry Eye Questionnaire'

**Source**	**df**	**MS**	**F**	**P' values**
Within subjects factor (Assessments)	1	0.133	3.221	0.075
Between subjects factor (Groups)	1	8.326	15.369	0.001
Interaction (Assessments and Groups)	1	7.381	178.607	0.001
Error (Within subjects factor)	115	4.133		
Error (Between subjects factor)	115	0.542		

Greenhouse-Geisser epsilon = 1.000, hence Sphericity Assumed

## Discussion

In the present single blind, randomized, prospective trial 291 persons working in a software company were evaluated for self-rated symptoms of visual discomfort. They were randomized as yoga (YG) and wait list (WL) control groups. Both groups showed comparable discomfort at baseline. At the end of sixty days the YG group showed decreased scores, whereas the WL group showed an increase in visual discomfort.

Visual discomfort in professional computer users is contributed to by various factors such as lighting, glare, display quality, ergonomic positioning of the monitor and regularity of work breaks [6]. The symptom which largely contributes to subjectively rated visual discomfort is 'dry eye'. Dry eye is itself contributed to by various factors, including certain diseases (e.g., Sjögren's syndrome, use of certain medication (e.g., anti-histaminics), gender (being more common in females)), and individual factors [17]. Individual factors include blink rate and completeness of blinking which significantly affect tear film dynamics and ocular surface health [18,19]. Blink rate especially has been shown to vary with the task performed [20]. The mean (± S.D.) rate of blinking was 22 (± 9) per minute under relaxed conditions, 10 (± 6) per minute while the subjects were reading a book at table level, and 7 (± 7) per minute while working at a video display terminal. Hence the frequency of blinking reduces while mentally alert and with gaze focused.

Specific yoga practices have been found to bring about physiological changes suggestive of 'alertful rest' [21]. This description was based on a simultaneous decrease in heart rate and oxygen consumption along with a reduction in peripheral cutaneous blood flow. Also the visual cleansing practices used in the present trial have been shown to facilitate visual perceptual sensitivity in terms of a decrease in optical illusion [22]. A reduction in anxiety has been found to be associated with better visual perceptual sensitivity [23]. A relaxed state (as described above) is associated with a higher frequency of blinking. Yoga practice has been associated with better self rated relaxation [24] as well as with physiological relaxation [25]. Hence the reduction in visual discomfort in the yoga group in the present study may be attributed to an improvement in the ability to focus while remaining relaxed which may have increased the blink rate.

In contrast to the yoga group the control group showed an increase in self rated visual discomfort. These differences between the groups could be due to psychological benefits that are reported with 'additional care' [26]. In the present study the frequent meetings which the yoga group had with the instructor could serve as additional care and may have contributed to the benefits seen in the yoga group. The absence of this psychological support and the yoga practice in the control group may have contributed to increased visual discomfort at follow-up.

A main limitation of the study is that well recognized objective indicators of visual discomfort (especially dryness) were not measured. It would have been ideal to have carried out a semi-quantitative estimation of the superficial lipid layer or have measured the tear breakup time [27]. However another variable which is an objective indicator of VDT related fatigue was measured in these subjects, and the results were reported elsewhere [28]. This is the critical flicker fusion frequency (CFF), which is the flicker frequency rate beyond which one can no longer perceive the flicker. Flicker related changes in the visual system from working at a cathode ray tube (CRT) computer screen have also been measured [29]. A group of subjects worked for 3 hours at simulated CRT displays with different flicker rates. The CFF was found to decrease. A similar result was obtained when the effect of performing the same task on a CRT was compared with the performance on a back slide projection system (BPS) [30]. The CFF of the group decreased after working on the CRT computer screen while it did not change when working on the BPS. These studies suggest that the visual system possibly gets fatigued as a result of viewing supra-threshold flicker.

In the subjects of this study critical flicker fusion frequency was measured using a standard electronic apparatus [28]. Each subject was assessed in 10 trials (5 each, ascending and descending, given alternately). The frequency of flicker for the ascending trials was gradually increased from a minimum of 8 Hz, with 1 Hz increments, till the subjects reported that the light appeared "fused" or steady. This was the fusion threshold. For descending trials, the frequency was gradually reduced (1 Hz per step) from 49 Hz, till the subject perceived the stimulus as "flickering". This was the flickering threshold. The average value of the ascending and descending trials was used for statistical analysis.

After sixty days the yoga group showed an increase in CFF from a group average (± SD) of 31.8 (± 2.6) at baseline to an average of 33.6 (± 2.5) after sixty days. In contrast, the wait list control group showed a decrease in CFF, from a group average of 32.5 (± 2.5) at baseline to a group average of 31.4 (± 2.5) at the end of sixty days. Hence the yoga group showed an average increase of 1.8 Hz in the CFF, compared with an average decrease of 1.1 Hz in the wait list control. This may suggest that the wait-list control group might have remained prone to visual fatigue, whereas the yoga group was not.

These results suggest that sixty days of yoga practice may have reduced visual fatigue based on the self-rated symptoms presented in this study and the CFF findings reported earlier [28]. However other factors may have influenced the subjective assessment of visual dryness. For example, certain personality traits were reported to be higher in contact lens wearers who had dryness of the eyes [31]. This study subjectively evaluated personality traits using the Yatabe Guilford Personality Test. No personality assessment was carried out in the participants studied here, which can be considered a limitation of the study. Also, it has been shown that for yoga practice to be effective participants should be motivated to learn and practice yoga [32]. Hence it would also have been useful to assess levels of motivation in the yoga group and correlate them with the reduction in self-rated visual discomfort which was found.

## Conclusion

The results of the present study suggest that a combination of yoga techniques practiced for 60 days improves self-rated visual discomfort in computer professionals. In contrast, the wait list control group who continued with their usual routine showed an increase in self-rated visual discomfort. Hence the practice of yoga can be a potential non-pharmacological intervention for visual discomfort related to working at visual display terminals (VDTs).

## Competing interests

The principal author and four co-authors declare that they have no competing interests.

## Authors' contributions

ST conceived and designed the study and prepared the manuscript. NKV participated in the conception and design of the study and in compiling the manuscript. MD co-ordinated the project and supervised the intervention and data collection. RD participated in the recruitment of subjects, data collection and assisted in statistical analysis. MNK carried out data extraction and analysis. All authors read and approved the final manuscript.
